# Subjective well-being in informal caregivers during the COVID-19 pandemic

**DOI:** 10.1515/med-2023-0739

**Published:** 2023-06-16

**Authors:** Daniela Alves Guedes, Nadirlene Pereira Gomes, Amâncio António de Sousa Carvalho

**Affiliations:** Grouping of Health Centres Tâmega I, Baixo Tâmega, Marão Várzea Personalised Health Care Unit, Amarante, Portugal; Nursing School, Federal University of Bahia, Salvador, Brazil; Department of Health School, University of Trás-os-Montes e Alto Douro/Nursing School, Vila Real, Portugal; CIEC – Research Centre on Child Studies, University of Minho, Braga, Portugal

**Keywords:** subjective well-being, informal caregivers, COVID-19, community health nursing

## Abstract

The study of subjective well-being (SWB) is important as it is related to the reduction of morbidity and mortality, with the maintenance of functionality and autonomy in the elderly population. The impact of the formative intervention on the SWB of informal caregivers (ICGs) during the pandemic crisis of COVID-19 was analyzed. This study is a quasi-experimental single-group, longitudinal study with a sample of 31 ICGs and their dependents. A form was used for data collection, and data processing was performed using IBM SPSS (Statistical Package for the Social Sciences), using descriptive statistics and inferential statistics. Of the total sample, the majority were female (90.3%). The difference between the mean of positive affection and negative affection at Moment 1 (M1) was –0.0581 ± 0.71590 and 0.04645 ± 0.53326 at Moment 2 (M2). The mean rank ordering of the difference between the two types of affection differed significantly between M2 and M1 (Wilcoxon: *p* < 0.000), with that of M2 being higher than M1 (16.93 > 2.50). The formative intervention, within the scope of community nursing, had a significant impact on increasing the SWB of the ICG in this sample. This study may contribute to improving the SWB of ICG and their dependents.

## 1 Introduction

The increase in average life expectancy, combined with a sharp fall in fertility rates, has led to an acceleration of population aging worldwide. These demographic changes have brought undeniable consequences for health, including an increase in the prevalence of chronic diseases, also causing an increase in the demand for health services by this population group, with an increasing trend in recent decades [1].

In Portugal, as in many other countries, the scenario is not different, with an increasingly notorious aging population. Indeed, it is the third country in Europe with the highest aging rate, according to the most recent statistics, referring to the year 2018 [2].

Older people with functional disabilities associated with chronic physical diseases, cognitive, mental or emotional, and motor diseases are recognized by the World Health Organization (WHO) as vulnerable or dependent. The same author states that the Council of Europe considers dependent as individuals who, for reasons associated with the reduction or even lack of some functional capacity, need to be helped to perform daily living activities, implying the presence of at least another person to support them. Such difficulties are classified into two categories: basic activities of daily living and instrumental activities of daily living. The first category includes self-care tasks such as dressing, eating, personal hygiene and moving around. The second includes the ability to perform activities necessary for personal and social development: participating in the community, performing practical tasks such as shopping, paying bills, keeping social appointments, using means of transportation, cooking, communicating, taking care of one’s health and maintaining one’s integrity and safety [3].

It should be noted that getting old is not synonymous with disease, disability or dependence, but it will not always be possible to reach this stage of life in perfect physical and mental health conditions. As the years pass by, there is an increasing probability of the individual acquiring a chronic and/or degenerative disease typical of the aging process itself, conditions which may incapacitate him/her and make him/her dependent, which will directly interfere with his/her autonomy, bringing damages to the domains related to his/her self-control and self-sufficiency [4].

Faced with this situation of dependence, the need for informal care emerges. According to the literature consulted, gerontology denominates informal caregivers (ICGs) as people who are part of the family nucleus, nucleus of friends, neighbors or other people from the community who, on a voluntary basis, without specific training and remuneration, exercise the role of ICG at home [5].

The task of informal care largely exceeds the simple assistance to the person who is incapacitated. According to Castro et al., ICGs are the main actors responsible for ensuring the well-being of family members who are dependent at home, besides being considered an added value for the National Health System, since they can complement, with their work, that which public health services, for whatever reasons, cannot meet in a timely manner [4].

Given the changes that have been emerging, Portugal has sought to provide an adequate response, namely through the creation of the ICG statute, approved as an annex to Law no. 100/2019, of 6 September. This statute was approved in Portugal in July 2019, being quite recent, aiming to improve the conditions and promote their well-being [6].

Performing the complex task of ICG may lead to uncomfortable and exhausting demands, which may affect their personal life. The experience of all these phenomena experienced in the exercise of the ICG activity is called burden in the literature, which may be of two types: objective burden and subjective burden [4].

Objective burden is related to the caregiving task and refers to time restriction, increased physical effort, economic expenses, changes in personal life, family relationships, and physical and mental health, and is related to the concrete events and activities that lead to changes in the caregiver’s life, being observable and quantifiable. In turn, the subjective burden is based on the caregiver’s particular understanding of the effects of the care task. This dimension of burden becomes evident when the ICG begins to manifest changes and damage in their psychological well-being, such as the feelings, attitudes and emotional relationships of the caregiver to the experience of caring and may contain indicators that assess the objective burden [5].

The ICGs who find themselves involved in care provision without prior preparation and due to lack of social support for this task are forced to give up their jobs, social and even family life. This activity is often carried out without the help and recognition of other family members, which generates a high degree of burden and abdications. This burden is associated with emotional exhaustion (emotional stress, depression) and physical exhaustion (chronic illnesses), in addition to social isolation, family conflicts, and financial difficulties [7].

Under normal circumstances, caregiving can be complex and potentially stressful. While offering some benefits, caregiving can also compromise the health and well-being of caregivers. Caregivers, both short and long term, also reported worse mental health and greater fatigue than non-caregivers. The COVID-19 pandemic has the potential to exacerbate the potential stresses of family caregiving [8].

This phenomenon of ICG burden has been greatly aggravated by the pandemic situation caused by Covid-19, which was decreed by the World Health Organization (WHO) in March 2020. In a desperate attempt to contain the spread of the virus and the spread of the disease Covid-19 causes, governments around the world have taken unprecedented measures. Entire cities, regions, and countries were isolated; travel was banned; schools and universities were closed; shops were beginning to run out of stock; and all economic, cultural, and social activities were stopped. Never before in modern history has a health problem had such an overwhelming impact on the society. Health (or rather the threat of health problems) has become the predominant concern that takes precedence over all other issues, causing health in all policies to become a reality, although not in the way it was intended [9].

According to Batello et al. [10], during the pandemic period, the preventive measure of social distancing caused a change in the routine of the person being cared for and required increased attention from the caregiver to prevent the contagion by the coronavirus, so they tend to have work overload. In addition, this whole scenario causes physical, emotional, and relational exhaustion in ICGs, which may compromise their health and the act of caring.

In turn, another study [11], based on a study including several European countries, states that large proportions of caregivers have experienced an increased burden and stress-related symptoms like trouble sleeping since the outbreak of the pandemic. Furthermore, ICGs frequently reported worsened physical and mental health, such as being depressed or anxious as well as feeling more socially isolated and lonely. Dellafiore et al. [12], in a systematic review on the consequences of the pandemic of COVID-19 on ICGs, identified two major themes: family caregivers' COVID-19-related stress and (Mal)adaptive strategies to the “new” normality.

In order to standardize the Covid-19 guidelines, the WHO has produced a guide with all the guidelines, which almost all countries worldwide have followed as a reference. This organization has prioritized the evaluation of approaches to care, using appropriate personal protective equipment (PPE), in order to prevent further contagion [13].

The Directorate-General for Health (DGH) in Portugal produced a manual with general measures for the prevention and control of COVID-19, which aimed to present the general measures to be adopted by all, being completed by several volumes with specific measures to be adopted in different contexts, based on the principles of evidence and scientific knowledge [14].

Sequeira reports that the biggest problem present in most ICGs focuses on the domain of information that lacks knowledge inevitably impacts on the recognition of early symptoms, delaying their integration into the care network and decreasing the possibilities of early intervention [5].

A study conducted in Portugal showed that the lack of training and technical ability on the part of the ICG were factors that implied insecurity to perform the care of the frail elderly [15].

The empowerment of the ICG to perform the role of caregiver to the person with dependence is a specific intervention of the health professionals involved in the care process. In this empowerment process, health professionals have an essential role. Through their empowerment process, it is possible to reduce healthcare costs and improve the quality of life of the service user and the ICG, their mental health, as well as their satisfaction with care. The contribution of caregivers will only be possible if they are given learning and training opportunities, as well as accessible and relevant support [16].

Some Portuguese authors address the construct of subjective well-being (SWB) based on the theory of Wilson (1967), one of the first authors to study this concept, stating that Wilson studied two hypotheses of well-being. In the first hypothesis, he related the concept of satisfaction and happiness, from a bottom-up perspective to immediate satisfaction of the needs that produce happiness. In the second, he related well-being to the degree of satisfaction necessary to produce happiness, originating from the top-down perspective [17].

Later, in the 1970s, the construct of well-being appeared associated with the concept of health, especially mental health, including positive dimensions such as SWB, perceived self-efficacy, autonomy, competence, self-actualization of intellectual and emotional potential, referring to SWB as a dimension of mental health [17].

Matamá et al. also argue that SWB requires self-assessment, i.e., this construct can only be observed and reported by the subject him/herself, considering SWB to be the assessment that people make of their own lives. They also add that the SWB can also be considered a positive dimension of health, which encompasses other concepts such as quality of life, positive affect, and negative affect, being seen as a broad set of phenomena that include emotional responses, domains of satisfaction, and global judgments of satisfaction with life [17].

Emotions are extremely important in people’s lives and assist in the choice of adaptive responses to face the difficulties of daily life, known as coping strategies, in addition to preserving social bonds and personal well-being. The process of choice that involves understanding, balancing, and deciding which emotions to feel and express is called emotional regulation. Acts of conscious regulation require the person to identify their desirable affective states and engage in some type of action, thought or behavior to adaptively cope with the momentary emotional experience. The ability to regulate positive emotions is of particular relevance to adaptation and mental health, as it strengthens attention, cognition, and creativity, increasing well-being, the quality of social relationships and work performance [17].

Concerning well-being and psychological well-being, several theoretical models have been proposed and developed, contributing to their definition, operationalization, and, consequently, assessment. The concept of SWB encompasses a cognitive component, formed by people’s reflective judgments about their satisfaction with life and an affective component, formed by emotional experiences, with a predominance of positive or pleasant emotions over negative or unpleasant ones [18].

Lima and Morais state that SWB is defined by individual and subjective evaluations that include the cognitive judgment of satisfaction with life and emotional reactions to life events. This construct for bottom-up theories is explained by the influence of external factors, situations, and variables; while in top-down theories, subjective variations, such as personality traits, are emphasized in the determination of well-being [19].

There are two main approaches to the concept of well-being: the hedonic and the eudaimonic perspectives. SWB is framed in the hedonic perspective, which refers to personal evaluations of the level of satisfaction with life, including personal analysis of the frequency with which positive and negative emotions are experienced in daily life. The eudaimonic perspective is based on psychological well-being, a construct that is not part of our object of study in this article [17].

The concept of SWB has received increased attention over the past few decades and basically consists of three components: positive affect, negative affect, and satisfaction with life [20].

Positive affects concern how enthusiastic, active, alert, jovial, self-confident, and caring a person is feeling. It refers to pure hedonic contentment, experienced at a given moment as a state of attentiveness and activity. On the other hand, negative affects refer to the general dimension of distress and dissatisfaction, a discomfort, including aversive mood states such as anger, guilt, disgust, and fear. Therefore, a high SWB means high rates of positive affects and satisfaction with life and low rates of negative affects [21].

Affects are the immediate response that a person has to a stimulus or event. They are based on the sensation of arousal, which allows us to evaluate the event as pleasurable or painful for the subject. Affects actively interfere in the way the subject sees his/her own life and the people around him/her. In this way, people with a higher prevalence of positive affect tend to feel more happy in their daily activities and those with more negative affect tend to look at daily life in a negative and sad way [21].

There are three aspects of SWB that are important to be highlighted: the first is subjectivity, in which well-being resides within the individual’s experience; the second consists of understanding that well-being is not only the absence of negative factors but also the presence of positive factors; the third emphasizes that well-being includes a global measure instead of only a limited measure of an aspect of life [20].

The term SWB is characterized by subjectivity in relation to the self-assessment of satisfaction with life, not only being identified by positive affections, such as joy, pleasure, and optimism, but also by the absence of negative affections. This construct is related to the reduction of morbidity and mortality, with the satisfactory maintenance of functionality and autonomy of the elderly population. Hence, it is important to study this phenomenon in dependent elderly people [22].

SWB has both short- and long-term impacts on people’s lives, and this is an important variable for the mental health of individuals. Thus, practices aimed at increasing SWB levels have positive impacts on the success of individuals, as well as on society as a whole. SWB is related to primary prevention, regarding the work of identifying negative factors, before something negative happens, seeking to prevent the disease [21].

A longitudinal type study with a sample of 22,576 patients aged 50 years or older, with coronary artery disease, by means of generalized estimating equations, showed that despite being an asymptomatic disease, patients with higher blood pressure had higher chances of reporting SWB levels below the ideal. It also showed that female patients, of the black race, with a history of smoking, diabetes, myocardial infarction, stroke, and cancer, also have higher chances of reporting low levels of SWB [20].

This study aims to analyze the impact of a formative intervention on the SWB of ICG during the pandemic crisis of COVID-19.

## 2 Methods

This is a quasi-experimental, single-group, longitudinal study with a quantitative approach [23]. The following inclusion criteria were used to define the population: (i) being an ICG of a dependent person in the geographical area of the Community Care Unit of the city of Amarante (Portugal) and (ii) being an ICG for at least half a year. After the application of the inclusion criteria, the target population was composed of 31 ICGs of dependent people, registered in the health units of Amarante in the year 2020/2021. The exclusion criteria were as follows: (i) the dependent person had died during the study and (ii) the ICG was on sick leave during the same period. As no ICGs were excluded, the sample coincided with the accessible population, which consisted of 31 ICGs. In this study, no sampling technique was applied due to the small size of the population.

A form consisting of four parts was used for data collection: the first part aimed at characterizing the population in socio-demographic terms; the second part aimed at assessing the ICGs’ knowledge about the prevention of Covid-19 disease; the third part contained the SWB scale – PANAS (original version built by Watson et al., [24]), through which the positive affect and negative affect were assessed, which was adapted and validated for the Portuguese population by Galinha and Pais-Ribeiro [25]. This scale is a measure of the SWB, assessing on a 5-point Likert-type scale ranging from “not at all” or “very slightly” (1), “a little” (2), “moderately” (3), “quite a lot” (4), and “extremely” (5), and how respondents experience, at the moment, ten positive emotions and ten negative emotions. The Portuguese version of PANAS presents an internal consistency of 0.86 for the Positive Affect Scale and 0.89 for the Negative Affect Scale [25]. The scale allows for determining the SWB, resulting from subtracting the negative affects from the positive affects; the fourth part of the form is composed of the Checklist – Handwashing elaborated based on the manual of General Prevention and Control Measures COVID-19 of the DGS, to guide the observation of the ICG in handwashing. In the present study, we only used parts I and III.

All ethical procedures were followed, taking into account the respect for human dignity, the right to confidentiality, the protection of anonymity and data confidentiality. In order to conduct the study, a request for authorization to apply the data collection form was submitted to the President of the Ethics Committee of the Regional Health Administration of the North, with a favorable opinion, reference no. CE/2021/24, dated June 17, 2021. Data collection was performed by researchers at the home of each ICG, during a home visit, having previously requested informed consent. Data collection took place, from the first moment (M1), in the period from April 1 to 9, 2021 and the second moment (M2), in the period from April 19 to 30, 2021. Between M1 and M2 of data collection, from April 12 to 16, 2021, training interventions were performed at the ICGs’ homes on an individual basis due to the pandemic context that we were going through, which prevented a group approach on the correct use of the mask and knowledge about the signs and symptoms of Covid-19, preventive measures, and the importance of proper hand hygiene. The intervention consisted of two individual health education sessions at the home of each ICG due to the pandemic situation that the country was going through. At the end of these sessions, a flyer was distributed to reinforce the issues addressed and the questionnaire for evaluation. The overall objective was to empower ICGs with knowledge and skills on the infection of COVID-19 and increase SWB. Knowledge was imparted on the signs and symptoms of this infection, infection control, going shopping, hand hygiene, and use of PPE, with demonstration and training of these last two preventive measures.

Data were processed and analyzed using IBM Statistical Package for the Social Sciences (SPSS), and a database was built in which they were entered. In terms of descriptive statistics, the absolute and relative frequency and mode were calculated for all variables. In the case of ordinal variables, the order statistics (percentiles, deciles) were also requested, and in the case of scalar variables, the calculation of the measures of central tendency and dispersion was also requested. In the case of inferential statistics, parametric statistical tests were used (Student’s *t*-test and ANOVA) and when the assumptions for their use were not met, non-parametric tests were used (Mann–Whitney and Kruskal–Wallis). The Wilcoxon test was used to compare the ordination means between the two data collection moments (M1 and M2). A statistical significance level of 0.05 was considered for all analyses performed [26].

## 3 Results

From the total sample (*n* = 31), most of the ICGs were female (90.3%), belonged to the age group of 45–64 years (61.3%), had a marital status of married (67.7%), had between 1 and 2 children (64.5%), had first cycle education (38.7%), a non-active labor situation (74.2%), the degree of kinship with the dependent was of son/daughter (61.3%), and lived with the dependent (58.1%) (Table 1).

**Table 1:** Sociodemographic characterization of ICGs: Amarante, Portugal, 2021 (*n* = 31)

Variables		Af	Rf
Gender	Male	3	9.7
	Female	28	90.3
Age group	18–44 years old	5	16.1
	45–64 years old	19	61.3
	65 and more	7	22.6
Marital status	Single	6	19.4
	Married/living together	21	67.7
	Divorced	4	12.9
Number of children	No children	6	19.4
	1 to 2 children	20	64.5
	3 or more children	5	16.1
Academic qualifications	First cycle (4th year)	12	38.7
	Second cycle (9th Year)	7	22.6
	Third cycle (12th year)	9	29
	Degree/master’s/doctorate	3	9.7
Employment situation	Not active	23	74.2
	Active	8	25.8
Relationship with the dependent	Mother/father	1	3.2
	Wife/husband	6	19.4
	Son/daughter	19	61.3
	Son/daughter-in-law	1	3.2
	Others	4	12.9
Cohabiting with the dependent	No	13	41.9
	Yes	18	58.1

Legend: Af – absolute frequency; Rf – relative frequency.

With regard to the distribution of the responses of the ICG to the emotions of positive affect (Table 2), the emotions that contributed most to the positivity of affections were feeling determined, active, and proud, which obtained the percentage of responses, respectively, in the option “quite a lot,” 48.4, 54.8, and 16.1%, and in the option “extremely,” all with a percentage of 9.7%. On the other hand, the emotions that contributed least to the positivity of affect were feeling enchanted, pleasantly surprised, and enthusiastic, which obtained the percentage of answers in the option “not at all” or “very slightly,” respectively, 93.5, 90.3, and 74.2%.

**Table 2:** Distribution of the response options of ICGs to the items of the SWB scale – PANAS: Amarante, Portugal, 2021 (*n* = 31)

	Not at all or Very slightly (1)		A little bit (2)		Moderately (3)		Quite (4)		Extremely (5)	
Emotions	Af	Rf	Af	Rf	Af	Rf	Af	Rf	Af	Rf
Interested	9	29.0	11	35.5	4	12.9	5	16.1	2	6.5
Disturbed	5	16.1	13	41.9	5	16.1	8	25.8	0	0
Excited	14	45.2	13	41.9	3	9.7	1	3.2	0	0
Tormented	13	41.9	7	22.6	6	19.4	4	12.9	1	3.2
Pleasantly surprised	28	90.3	2	6.5	1	3.2	0	0	0	0
Guilty	30	96.8	1	3.2	0	0	0	0	0	0
Scared	7	22.6	9	29.0	8	25.8	6	19.4	1	3.2
Warm	16	51.6	11	35.5	1	3.2	3	9.7	0	0
Disgusted	24	77.4	2	6.2	2	6.5	1	3.2	2	6.5
Excited	23	74.2	5	16.1	2	6.5	1	3.2	0	0
Proud	19	61.3	4	12.9	0	0	5	16.1	3	9.7
Irritated	9	29.0	8	25.8	7	22.6	6	19.4	1	3.2
Enchanted	29	93.5	1	3.2	0	0	1	3.2	0	0
Remorse	29	93.5	1	3.2	0	0	1	3.2	0	0
Inspired	17	54.8	9	29.0	4	12.9	1	3.2	0	0
Nervous	4	12.9	7	22.6	7	22.6	10	32.3	3	9.7
Determined	2	6.5	3	9.7	8	25.8	15	48.4	3	9.7
Trembling	15	48.4	11	35.5	5	16.1	0	0	0	0
Active	0	0	4	12.9	7	22.6	17	54.8	3	9.7
Frightened	8	25.8	7	22.6	4	12.9	10	32.3	2	6.5

Legend: Af – absolute frequency; Rf – relative frequency.

In relation to negative affect, the emotions that contributed most to this were feeling nervous, frightened, and disturbed, which obtained a percentage of answers in the option “quite a bit,” respectively, of 32.3, 32.3 and 25.8%. The emotions that contributed least to the negativity of affect were feeling disturbed, nervous, and scared, with a percentage of answers in the option “not at all” or “very slightly,” respectively, of 16.1, 12.9, and 22.6%.

The mean difference between positive affects and negative affects at M1 and M2, before and after the intervention, was −0.0581 ± 0.71590 and 0.04645 ± 0.53326, respectively; the difference was higher at M2.

In the SWB categorization, positive affect and negative affect obtained the same percentage (48.4%), meaning that the sample is almost equally divided between both types of affects (Table 3).

**Table 3:** Categorization of the SWB reported by ICGs: Amarante, Portugal, 2021 (*n* = 31)

Categorization of SWB	Af	Rf (%)
SWB negative	15	48.4
SWB neutral	1	3.2
SWB positive	15	48.4
Total	31	100

Legend: Af – absolute frequency; Rf – relative frequency.

The mean rank order of the difference between positive and negative affects of the ICGs differed significantly between Moment 2 and Moment 1 (Wilcoxon: *p* < 0.000), with the mean rank order of the difference of Moment 2 (16.93) being higher than that of Moment 1 (2.50). This means that the training action increased the SWB of ICGs. Of the 31 ICGs who participated in the training action, 29 increased their SWB and 2 ICGs decreased their SWB (Table 4).

**Table 4:** Results of the Wilcoxon test between the mean rank difference of positive affects and negative affects at Time Point 1 and Time Point 2: Amarante, Portugal, 2021 (*n* = 31)

Variables	Classification	*n*	Mean rank	Test value	*p* value
Difference between the mean rank of positive affects and negative affects M2 – difference between the mean of positive affects and negative affects M1	Negative	2	2.50	Wilcoxon = −4.770	0.000
	Positive	29	16.93		
	Draw	0			

Legend: *n* – absolute frequency; *p* – probability.

## 4 Discussion

In Portugal, in 2019, there were 1,059,012 people who provided informal care and most of them were women (65.4%); the largest age group was between 25 and 64 years old (70.98%) and their literacy level was basic education or first cycle (53.7%), which is consistent with our study [27].

According to Ferreira et al., there is a predetermination of who will be the caregiver, once there is a social expectation that it is the woman who assumes this role since taking care of the family and performing domestic chores are functions considered “naturally” feminine. Thus, the caregiving role assigned to women seems to be the result of historical and social construction, in which girls are taught from childhood to perform caregiving tasks, creating the expectation that they will exercise the role of caregiver, when necessary, throughout their lives. Therefore, one can designate culture as a guiding element for the choice of who will assist the elderly in their aging process [7].

With regard to work status, the results of our study are in line with the results presented in the Autumn Report (2018), in which the majority of ICGs (54%) were inactive, with slightly more than half being in retired status [28].

A study was conducted in the municipality of São Carlos (São Paulo, Brazil), with 44 caregivers, which aimed to assess and compare the sociodemographic characteristics, depressive and anxiety symptoms, and perceived stress of formal and ICGs of older people with Alzheimer’s disease; the majority of caregivers were son/daughter (65.4%), as in the present study. The fact that most of these caregivers were sons/daughters reinforces the idea of the feeling of obligation to care for these family members or even the lack of resources [29].

Both the mean of the difference between positive affects and negative affects and the mean rank of the difference between these affections are higher in M2 after the formative interventions, which means that they had a positive impact on SWB, significantly increasing positive affects. Of the 31 ICGs who composed our population, 29 (93.5%) obtained a positive difference between affections and, in only two ICGs, this difference was negative. The formative interventions on the preventive measures of COVID-19 thus seem to have contributed to improving the SWB of the ICG. As mentioned by Batello et al., who developed a reflexive analysis conducted in Brazil, these actions are necessary and urgent in order to identify and address problems and ensure the quality and continuity of care for dependent people [10].

These results corroborate those obtained by a study conducted in the city of Tomsk (Russia), in a sample of 119 older people participating in social engagement projects, which increased the participants’ SWB [30], highlighting the difference between the population in question and the fact that these projects were more comprehensive than ours.

Also, a systematic review and meta-analysis study showed small to moderate effects on subjective well-being and suggested that individualized multicomponent interventions for caregivers may be one of the ways to promote their well-being [31].

The main limitations of this study may be the fact that there was no control group, and there may have been interference from extraneous variables in the impact of the interventions performed.

This study may have implications for the professional practice of the nursing team, who provide care to these families, playing the role of ICG, to one of the elements of the family system with dependence, raising their awareness about the support that they can provide and the importance of training ICGs, for the prevention of COVID-19 and other pathologies, protecting their most fragile family members, for the improvement of their own SWB and the care provided to the dependent people in their care.
